# Integrative Multi-Omics Analysis Reveals Molecular Signatures of Recurrence in Paired Primary and Recurrent High-Grade Serous Ovarian Cancer

**DOI:** 10.3390/ijms27020948

**Published:** 2026-01-18

**Authors:** Min-A Kim, Johyeon Nam, Ha-Yeon Shin, Jue Young Kim, Anna Jun, Hanbyoul Cho, Mi-Ryung Han, Jae-Hoon Kim

**Affiliations:** 1 Department of Obstetrics and Gynecology, Gangnam Severance Hospital, Yonsei University College of Medicine, Seoul 06273, Republic of Korea; makim302@yuhs.ac (M.-A.K.); hayeon37@yuhs.ac (H.-Y.S.); forsythia410@yuhs.ac (J.Y.K.); anna1414@yuhs.ac (A.J.); hanbyoul@yuhs.ac (H.C.); 2 Division of Life Sciences, College of Life Sciences and Bioengineering, Incheon National University, Incheon 22014, Republic of Korea; j.nam.981214@gmail.com; 3 Institute for New Drug Development, College of Life Sciences and Bioengineering, Incheon National University, Incheon 22014, Republic of Korea

**Keywords:** high-grade serous ovarian cancer, recurrence, transcriptomics, proteomics

## Abstract

High-grade serous ovarian cancer (HGSOC) is the most prevalent and aggressive form of epithelial ovarian cancer and is characterized by high recurrence rates and poor clinical outcomes. In this study, we identify molecular signatures associated with recurrence by conducting integrative transcriptomic and proteomic analyses on paired primary and recurrent HGSOC tissues from 34 patients. RNA sequencing and proteomic profiling revealed 185 differentially expressed genes (DEGs) and 36 differentially expressed proteins (DEPs) linked to recurrence. Pathway enrichment and Ingenuity pathway analyses highlighted the involvement of immune cell trafficking, cell signaling, and MAPK pathway activation in recurrent tumors. A survival analysis identified seven DEGs that correlated significantly with recurrence-free survival; among them, *IL7R*, *IRF8*, and *PTPRC* were upregulated in recurrent tumors and associated with poor prognosis, and *NSG1* was downregulated and linked to favorable outcomes. Immunohistochemistry validated the differential expression of these markers at the protein level. The proteomic analysis demonstrated that recurrent tumor-specific DEGs are functionally linked to MAPK signaling. Co-expression analyses revealed dynamic regulatory interactions between the DEGs and DEPs, suggesting context-dependent molecular shifts during recurrence. This integrative multi-omics approach reveals that key molecular alterations underlie HGSOC recurrence and identifies *IL7R*, *IRF8*, *PTPRC*, and *NSG1* as potential prognostic biomarkers and therapeutic targets. Our findings provide a foundation for targeted strategies to improve outcomes for patients with recurrent HGSOC.

## 1. Introduction

Epithelial ovarian cancer (EOC) has the highest mortality rate among all gynecological cancers [1]. This cancer type comprises five distinct histological subtypes; the most common is high-grade serous ovarian cancer (HGSOC), which accounts for 70% of invasive EOCs. The other subtypes are low-grade serous (<5%), endometrioid (10%), clear cell (10%), and mucinous (3%) [2,3]. The standard treatment for HGSOC, the most aggressive subtype of EOC, is optimal debulking surgery followed by adjuvant chemotherapy with a platinum-based cytotoxic agent and paclitaxel [4,5]. However, 70–80% of patients with advanced HGSOC experience a recurrence within 14–24 months after first-line treatment, primarily due to cancer metastasis and the development of drug resistance [6,7,8,9,10,11,12]. Discovering effective therapeutic targets continues to be a key unresolved objective in the field [13,14,15].

Considerable research efforts have been directed toward elucidating the molecular and genetic characteristics of HGSOC to facilitate the development of more effective and personalized treatment strategies. Despite major advances in therapeutic approaches during the past two decades, including traditional cytotoxic drugs and emerging agents with diverse mechanisms such as VEGF inhibitors, PARP inhibitors, and anti-PD/PD-L1 inhibitors, treating HGSOC remains highly challenging [16,17,18]. Therefore, gaining a deeper understanding of the underlying biology of this disease is essential to the development of innovative and more effective therapeutic strategies [19].

Recent years have seen a notable increase in studies using whole-genome sequencing, whole-transcriptome sequencing, and single-cell analyses to analyze public datasets [20,21,22,23]. However, little research has compared RNA sequencing results between recurrent and primary ovarian cancer using actual tissue samples from the same patients. This omission is primarily because obtaining recurrent tumor samples longitudinally from the same patient is challenging in real-world clinical practice due to the lack of established consensus on performing secondary debulking surgery.

In this study, we conducted a comparative transcriptomic analysis using formalin-fixed paraffin-embedded (FFPE) samples from paired primary and recurrent HGSOC tissues collected longitudinally from individual patients in real-world clinical settings. To further elucidate downstream effects, we performed complementary proteomic analyses on fresh-frozen tissues, which enabled us to gain more detailed insights into the molecular mechanisms associated with recurrence. Our objective was to identify clinically relevant differentially expressed genes (DEGs) associated with disease recurrence in HGSOC.

## 2. Results

### 2.1. Patient Characteristics

The clinicopathological characteristics of the 34 enrolled patients are summarized in Table 1. The median age at diagnosis was 53 years (range 41–69), and the median serum CA125 level at diagnosis was 639.5 U/mL (range 50.0–20,388.1). Most patients were diagnosed at FIGO stage III (82.4%), with high-grade tumors (76.5%, G3), consistent with the typical presentation of HGSOC. BRCA status was available for 27 patients, and BRCA1 and BRCA2 mutations were identified in 11.8% and 5.9% of cases, respectively. Expert pathologists reviewed all tissue samples to confirm the histological subtype and tumor content. Samples with inadequate tumor proportion or poor RNA/protein quality were excluded from the analyses.

### 2.2. Gene Expression Alterations Between Matched Primary and Recurrent HGSOC

**Figure 1:**
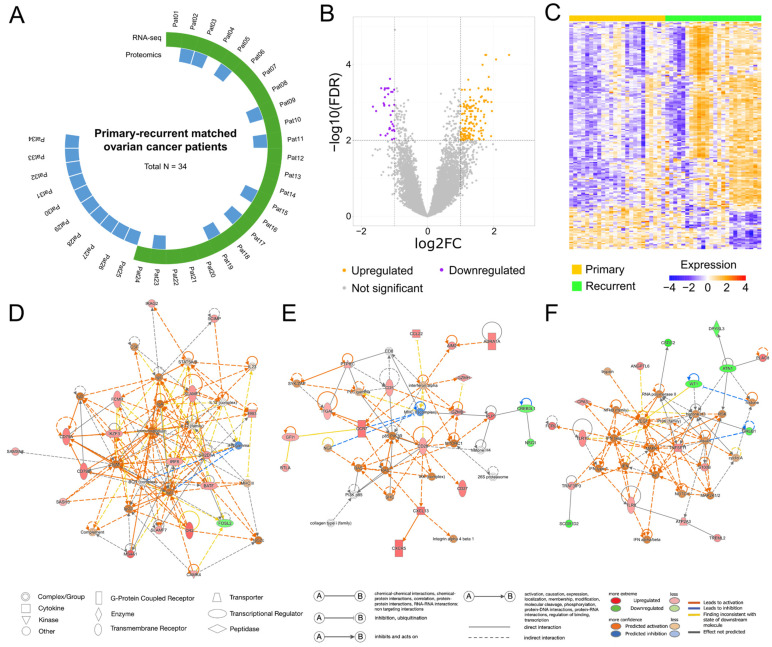
Transcriptomic alterations and molecular interaction networks in matched, primary and recurrent high-grade serous ovarian cancer. (**A**) Schematic representation of the patient cohort included in this study. A total of 34 patients with matched primary and recurrent high-grade serous ovarian cancer samples were analyzed. Green segments indicate patients subjected to RNA sequencing, whereas blue segments indicate those analyzed by proteomics. (**B**) Volcano plot illustrating differentially expressed genes (DEGs) between primary and recurrent tumors. Genes with a false discovery rate (FDR) < 0.05 and absolute log_2_ fold change > 1 were considered significant. Genes upregulated in recurrent tumors are shown in orange, downregulated genes in purple, and non-significant genes in grey. (**C**) Heatmap showing expression profiles of DEGs across all samples. Columns represent individual tumor samples (primary, yellow; recurrent, green), and rows represent DEGs. Color intensity indicates relative expression levels, ranging from blue (low) to red (high). (**D**–**F**) Gene regulatory networks generated using Ingenuity pathway analysis (IPA) based on the DEGs identified between primary and recurrent tumors. Nodes represent molecules, with red and green indicating observed upregulation and downregulation, respectively. Orange and blue nodes denote molecules predicted by IPA to be activated or inhibited. Solid and dashed lines indicate direct and indirect molecular interactions, respectively, based on curated literature and experimental evidence.

To identify potential biomarkers of HGSOC recurrence, we analyzed differences in the expression of protein-coding genes between matched primary and recurrent tumor samples. Using the criteria of a false discovery rate (FDR) < 0.01 and absolute log2 ratio of fold change (|log2FC|) > 1, we identified 185 DEGs, of which 151 (81.6%) were upregulated and 34 (18.4%) downregulated in recurrent HGSOC (Figure 1B, Supplementary Table S1). The gene expression patterns of the two groups are visualized in a heatmap (Figure 1C). These findings highlight a significant difference in gene expression patterns between primary and recurrent HGSOC.

### 2.3. Ingenuity Pathway Analysis Reveals Key Molecular Networks in Recurrent HGSOC

An Ingenuity pathway analysis (IPA) was conducted to explore the molecular networks and biological functions associated with the 185 DEGs, and it revealed 11 networks with scores higher than 20 (Supplementary Table S2). The highest-scoring network (score = 34) was associated with cell morphology, immunological diseases, and organismal injury. In that network, the algorithm focused on activating the BCR complex and several transcriptional regulators, including *IRF8*, *BATF*, and *IKZF3*. Based on that observation, the activation of multiple complexes, including various immunoglobulin complexes and the STAT5A/B complex, was predicted to be higher in recurrent HGSOC than in primary HGSOC (Figure 1D). The second-highest network (score = 34) was enriched for cell-to-cell signaling, hematological system development, and immune cell trafficking, and it had increased expression of the *CCR7*, *ITGAL*, *CD28*, *PTPRC*, *CD5*, *GZMB*, and *GZMH* genes. The pattern indicated the activation of RAS, mTORC1, p85-PIK3R, and NGF, alongside inhibition of the MHC II complex (Figure 1E). The third-highest network (score = 32) involved 17 focus molecules linked to endocrine, gastrointestinal, and metabolic disorders. That network predicted the activation of complexes such as MAPK, NOTCH, VEGF, and the NF-κB family, based on the observed expression of genes such as *TNFSF11*, *S100B*, *TLR10*, and *TLR8* (Figure 1F). Taken together, these findings imply that the functions of the DEGs were closely associated with the molecular characteristics of the cancer, highlighting their potential effects in cancer recurrence.

### 2.4. Pathway Enrichment Analysis Identifies Molecular Features Underlying Recurrence in HGSOC

**Figure 2:**
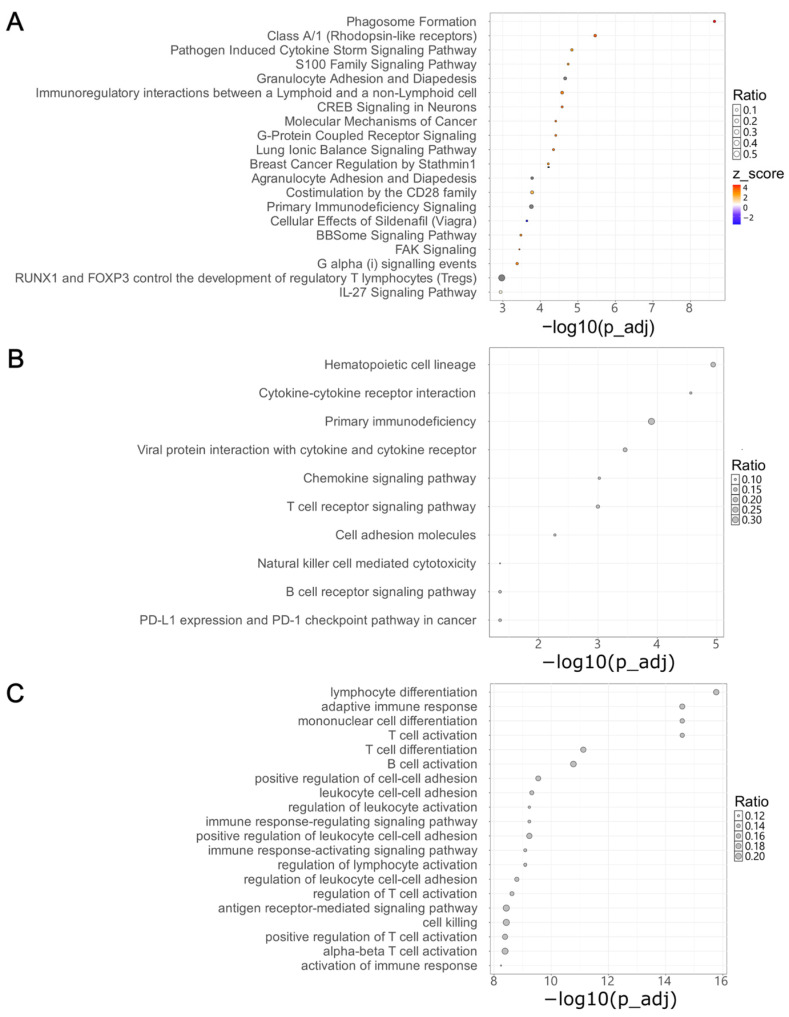
Pathway enrichment analysis of differentially expressed genes (DEGs) between matched primary and recurrent high-grade serous ovarian cancer samples. (**A**) Canonical pathway analysis based on the QIAGEN knowledge base. The top 20 significantly enriched pathways are shown. Dot color indicates predicted pathway activity: red, activated pathways; blue, inhibited pathways; and grey, pathways with undetermined directionality. (**B**) Over-representation analysis (ORA) of KEGG pathways. The top 10 significantly enriched pathways are shown based on overlap with differentially expressed genes. (**C**) ORA of Gene Ontology Biological Process (GO: BP) terms. The top 20 enriched biological processes are shown. Gene ratios on the *x*-axis represent the proportion of input DEGs associated with each pathway or process.

A canonical pathway analysis conducted with the IPA revealed 27 significant pathways associated with the DEGs (FDR < 0.01). The significant pathways mainly consisted of cell signaling pathways and pathways implicated in cancer mechanisms, such as molecular mechanisms of cancer and breast cancer regulation by stathmin 1 (Figure 2A). An over-representation analysis (ORA) was performed using the Kyoto Encyclopedia of Genes and Genomes (KEGG) database and Gene Ontology Biological Process (GO: BP) gene sets to validate these findings. In the KEGG analysis, 10 significantly enriched pathways (*p* < 0.05) were identified, including those involving cell adhesion molecules, natural killer cell-mediated cytotoxicity, and the PD-1/PD-L1 pathway in cancer (Figure 2B). The GO: BP analysis highlighted similar processes related to immune cell activation, cell adhesion, and cell signaling (Figure 2C). In summary, we identified significant gene expression differences between primary and recurrent HGSOC tissues, with DEGs enriched in pathways related to cell signaling and cancer mechanisms, providing potential insights into the molecular drivers of HGSOC recurrence.

### 2.5. Survival Analysis Identifies Prognostic DEGs in Recurrent HGSOC

**Figure 3:**
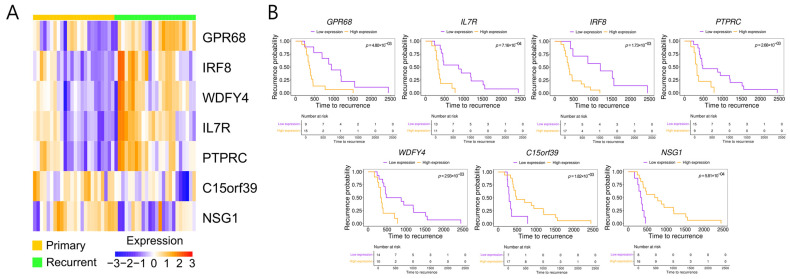
Differential expression and prognostic significance of candidate differentially expressed genes (DEGs) in primary and recurrent high-grade serous ovarian cancer. (**A**) Heatmap showing the expression levels of selected DEGs in primary and recurrent tumor samples. Columns represent individual tumor samples (primary, yellow; recurrent, green), and rows represent DEGs. Color intensity indicates relative expression levels, ranging from blue (low) to red (high). (**B**) Kaplan–Meier curves showing time to recurrence for patients stratified into high- and low-expression groups for each indicated DEG (*GPR68*, *IL7R*, *IRF8*, *PTPRC*, *WDFY4*, *C15orf39*, *NSG1*). Statistical significance was evaluated using the log-rank test, with *p*-values shown on each plot.

We performed a survival analysis using clinical data from the patients whose tissues we used to identify the DEGs significantly associated with HGSOC recurrence. That analysis revealed 11 DEGs associated with significant differences in recurrence-free survival between patients with high and low expression in their primary tumors (*p* < 0.01; Supplementary Table S3). Among them, we prioritized seven candidate DEGs based on their tumor-specific expression profiles and known or predicted prognostic relevance. We generated a heatmap using RNA sequencing data from the matched samples to illustrate the expression differences in the seven selected DEGs between primary and recurrent HGSOC tissues (Figure 3A). *GPR68*, *IL7R*, *IRF8*, *PTPRC*, and *WDFY4* showed higher expression in recurrent tumors, and *C15orf39* and *NSG1* were more highly expressed in primary tumors. The survival analysis revealed that high expression of *GPR68*, *IL7R*, *IRF8*, *PTPRC*, and *WDFY4* in the primary tumors was associated with poor prognosis, with the high-expression group showing worse outcomes than the low-expression group. In contrast, *C15orf39* and *NSG1* were linked to more favorable outcomes (Figure 3B). As *C15orf39* is currently an uncharacterized protein, the remaining six DEGs were selected for subsequent immunohistochemical (IHC) validation.

### 2.6. Experimental Validation of DEGs Between Primary and Recurrent HGSOC

**Figure 4:**
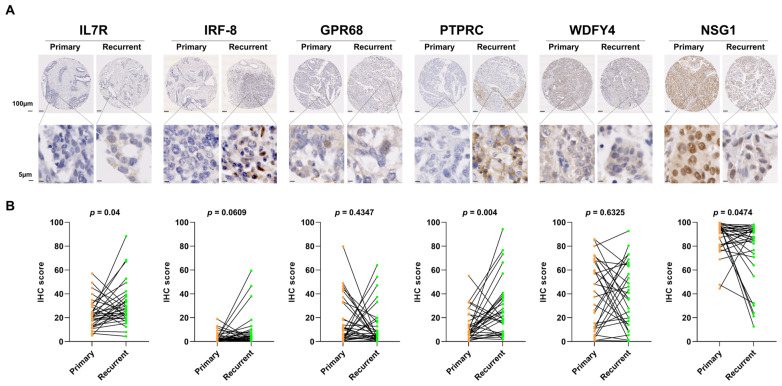
Differential protein expression of six candidate genes in matched primary and recurrent high-grade serous ovarian cancer tissues assessed by immunohistochemistry. (**A**) Representative immunohistochemical staining of IL7R, IRF8, GPR68, PTPRC, WDFY4, and NSG1 in tissue microarrays derived from paired primary and recurrent tumor samples. Scale bars represent 50 or 100 µm. Notable differences in expression were observed for IL7R, PTPRC, and NSG1 between matched tumor pairs. (**B**) Paired dot plots showing immunohistochemical staining scores for each of the six proteins. Black lines connect matched tumor pairs (primary and recurrent) from the same patient. Statistical significance of expression differences between primary and recurrent tumor samples was assessed using paired *t*-tests.

To validate the RNA sequencing results at the protein level, we performed IHC using a tissue microarray (TMA). The TMA consisted of paired primary and recurrent tumor tissues from 34 HGSOC patients, with patient information organized based on the primary tumor diagnosis. Importantly, this paired TMA cohort served as an independent validation set and had no patient overlap with the RNA-seq and proteomics cohorts. All the tissues included were of the serous subtype, and 91% of these patients were classified as stage 3 or 4. During the IHC process, some tissue cores were lost, leading to variation in the number of patients analyzed for each protein, as summarized in Supplementary Table S4. Protein expression was quantified using QuPath for IL7R (*n* = 32), IRF8 (*n* = 33), GPR68 (*n* = 32), PTPRC (*n* = 32), WDFY4 (*n* = 30), and NSG1 (*n* = 31). The protein expression levels of IL7R, PTPRC, and NSG1 were consistent with the RNA sequencing results. Specifically, IL7R and PTPRC were significantly upregulated in recurrent HGSOC tissues, whereas NSG1 showed reduced expression. These differences were statistically significant based on paired *t*-tests. Although the change in IRF8 expression did not reach statistical significance, it exhibited a trend of increased expression in recurrent tissues, similar to the RNA sequencing results (Figure 4). Therefore, the expression patterns of four of the six proteins (IL7R, PTPRC, NSG1, and IRF8) were consistent with the RNA sequencing findings, supporting the potential role of these proteins in HGSOC recurrence.

### 2.7. Co-Expression of DEGs and Their Regulatory Interactions with Differentially Expressed Proteins (DEPs) in Primary and Recurrent HGSOC

**Figure 5:**
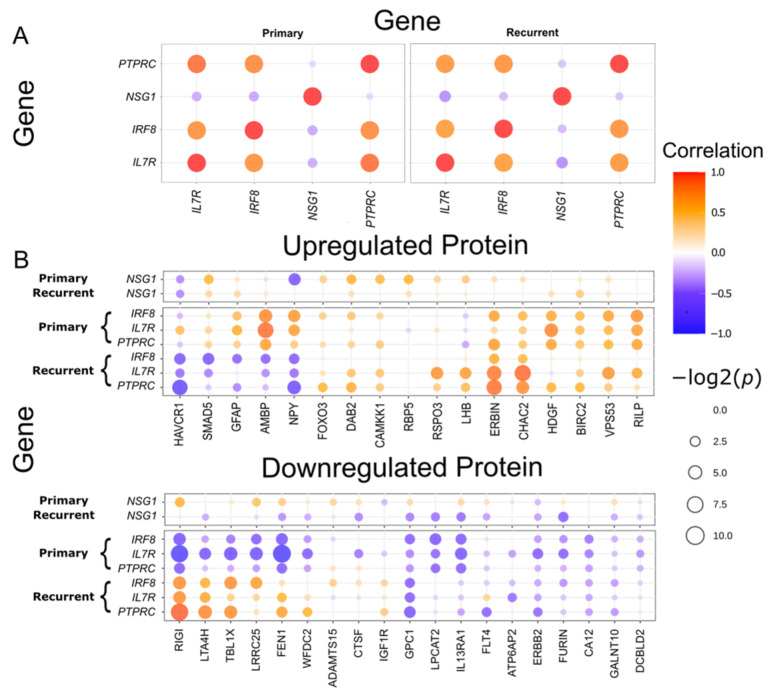
Co-expression relationships between differentially expressed genes (DEGs) and proteins in primary and recurrent high-grade serous ovarian cancer. (**A**) Dot plot showing co-expression patterns among four DEGs in primary and recurrent tumor samples. (**B**) Dot plot showing co-expression relationships between DEGs and differentially expressed proteins (DEPs) in primary and recurrent tumor samples.

We first examined the co-expression relationships among the four DEGs to investigate potential regulatory interactions. The expression levels of IL7R, IRF8, and PTPRC were significantly upregulated in recurrent tumors, compared with primary tumors, and correlated strongly with one another across both tumor types (*p* < 0.05; Figure 5A, Supplementary Table S5). Consistent with the survival analysis results, these three DEGs exhibited similar co-expression patterns with one another, whereas the primary tumor-specific DEG NSG1, which was downregulated in recurrent tumors, displayed an opposite expression trend. These findings suggest that IL7R, IRF8, and PTPRC are functionally linked or involved in a shared molecular pathway distinct from that of NSG1.

We performed a proteomic assay to further explore the biological significance of these candidate genes at the protein level. Among the 360 proteins that met our quality control criteria, 36 DEPs were identified between primary and recurrent HGSOC (*p* < 0.05), with 17 upregulated and 19 downregulated in recurrent tumors. To gain insight into potential regulatory interactions and downstream effects, we next examined the co-expression relationships between the DEGs and DEPs using data from the seven patients with both RNA sequencing and proteomic profiles (Figure 5B, Supplementary Table S6). Unlike the consistent co-expression patterns observed among DEGs across both tumor types, the DEG–DEP correlations exhibited divergent patterns between primary and recurrent HGSOC.

In primary HGSOC, the recurrent tumor-specific DEG IL7R exhibited a positive correlation with AMBP and HDGF and a negative correlation with FEN1, LRRC25, RIGI, and TBL1X (*p* < 0.05). Additionally, AMBP correlated positively with IRF8. Conversely, the primary tumor-specific DEG NSG1 did not exhibit significant associations with protein expression in primary tumors.

In recurrent HGSOC, the recurrent tumor-specific DEGs *IL7R*, *IRF8*, and *PTPRC* displayed significant correlations with seven proteins. Positive correlations were observed with CHAC2, ERBIN, and LTA4H, and negative correlations were identified with HAVCR1 and NPY. Co-expression between those DEGs and two DEPs (RIGI and TBL1X) was also detected. Notably, RIGI and TBL1X, both of which were downregulated in recurrent tumors, showed positive correlations with the DEGs in recurrent tumors, whereas they were negatively correlated in primary tumors. This reversal in correlation direction was unique to RIGI and TBL1X; the other five proteins maintained consistent patterns with the DEGs across tumor types (*p* < 0.05), suggesting a context-dependent shift in regulatory relationships during tumor progression. Similar to its behavior in primary tumors, NSG1 did not exhibit significant associations with protein expression in recurrent tumors. These findings highlight the dynamicity of DEG–DEP interactions across disease stages and suggest that the functional roles of recurrent tumor-specific DEGs are shaped by distinct molecular contexts in primary versus recurrent HGSOC.

To determine whether the DEPs were associated with molecular pathways predicted to be activated in our network analysis, we categorized them into functional gain and functional loss groups based on a comprehensive literature review. The functional gain group, which included CHAC2, ERBIN, LTA4H, HAVCR1, and NPY, was primarily involved in the RAS–RAF–MAPK signaling pathway. In contrast, proteins in the functional loss group were associated with a broader range of pathways, including the PI3K–AKT–mTOR pathway, the MAPK pathway, base excision repair, and NF-κB signaling (Supplementary Table S7). Collectively, our proteomic analysis suggests that recurrent tumor-specific DEGs are predominantly linked to the MAPK pathway through multiple mechanisms, underscoring their potential roles in HGSOC recurrence.

## 3. Discussion

In this study, we performed integrative transcriptomic and proteomic analyses of matched primary and recurrent HGSOC tissues from individual patients and identified distinct molecular alterations—highlighting key genes, proteins, and regulatory networks as potential biomarkers and therapeutic targets in ovarian cancer recurrence. Our RNA sequencing analysis identified 185 DEGs between primary and recurrent tumors, with 151 genes upregulated and 34 downregulated in recurrent HGSOC. Pathway enrichment analyses using IPA revealed molecular networks involved in immune cell trafficking and cell morphology regulation, which are critical for immune evasion and metastasis. A survival analysis incorporating patient clinical data further narrowed those possibilities to seven key candidate genes associated with recurrence: *IL7R*, *IRF8*, *PTPRC*, *NSG1*, *GPR68*, *WDFY4*, and *C15orf39*. Our IHC analysis to validate the protein-level expression of the selected genes confirmed significant upregulation of IL7R, IRF8, and PTPRC in recurrent tumors, whereas NSG1 exhibited a decreasing trend. Moreover, lower expressions of IL7R, IRF8, and PTPRC and higher NSG1 expression were associated with improved prognosis, highlighting their potential as prognostic biomarkers. Functional validation further demonstrated that IL7R, IRF8, and PTPRC contribute to tumor progression by promoting immune evasion, whereas NSG1 might have tumor-suppressive effects.

Previous studies analyzing matched primary and recurrent HGSOC tissues highlighted the dynamicity of the tumor microenvironment (TME), revealing profound alterations in immune composition, stromal interactions, and vascular remodeling that contribute to immune evasion and therapeutic resistance [24,25,26,27,28,29,30]. Recurrent tumors frequently exhibit enhanced immune evasion mechanisms, including upregulation of immune checkpoint molecules (e.g., PD-1/PD-L1, CD80/CTLA-4) and dysregulated immune cell trafficking, reflecting complex immunoregulatory processes [31,32]. Among immune cells in the TME, tumor-associated macrophages (TAMs) and regulatory T cells (Tregs) are pivotal in establishing an immunosuppressive niche [26]. Additional contributors to treatment resistance include cancer stem cells (CSCs) and autophagy, which are implicated in chemotherapy failure and disease relapse [30,33]. Furthermore, stromal and vascular components actively shape the TME; cancer-associated fibroblasts facilitate tumor proliferation, invasion, and immune evasion, while endothelial cells drive angiogenesis through VEGF signaling [34]. Although angiogenesis remains essential for tumor growth, recent evidence suggests that microvessel density and VEGF expression act more as supportive factors than direct drivers of disease progression—emphasizing the need for combination therapies that target multiple aspects of the TME [29]. Advances in multi-omics approaches have identified therapeutic strategies targeting the TME, such as anti-angiogenic agents (e.g., bevacizumab), TAM-targeted therapies, and immune checkpoint inhibitors (e.g., PD-1/PD-L1 blockade), that show clinical potential [24,35]. Nevertheless, resistance to immune-based therapies remains a challenge, particularly in recurrent tumors with high PD-L1 expression. Continued investigation is needed to refine these strategies, enhance patient stratification, and develop novel combinatorial therapies that effectively modulate the TME and improve clinical outcomes in ovarian cancer.

Our study highlights the importance of analyzing matched primary and recurrent HGSOC tissues to gain deeper insights into the molecular alterations that drive tumor recurrence. By characterizing these changes, we emphasize that a comprehensive understanding of the mechanisms underlying recurrence is crucial for developing targeted therapeutic strategies to disrupt its processes and improve patient outcomes. Among the genes we identified in this study, *IL7R*, *IRF8*, *PTPRC*, and *NSG1* have not been thoroughly explored or recognized for their relevance in ovarian cancer recurrence in previous studies. We found them to be notably associated with tumor recurrence and clinical prognosis, so their potential roles in HGSOC biology warrant further investigation.

Although IL-7 plays a critical role in enhancing anti-tumor immunity through its immunomodulatory effects, increasing evidence suggests that activation of its receptor, IL-7R, might paradoxically promote tumor progression by supporting tumor cell proliferation and facilitating immune evasion via TME modulation [36]. Due to its dual role in cancer biology, IL-7R has emerged as a potential therapeutic target, prompting efforts to exploit its signaling axis to improve the efficacy and safety of cancer immunotherapies [37]. IL-7 restores CD8^+^ T cell function by reducing exhaustion markers such as PD-1 and simultaneously promoting the expansion of Tregs, which can suppress anti-tumor immunity [36,38,39]. This dual effect suggests that IL-7R upregulation in recurrent HGSOC facilitates immune escape by disrupting the balance between cytotoxic and regulatory immune cell populations within the TME. Mechanistically, IL-7R signaling activates several key oncogenic pathways—including JAK/STAT5, PI3K/AKT/mTOR, Ras/ERK, and MAPK—that regulate genes involved in cell proliferation, apoptosis resistance, and immune modulation [36]. The inhibition of IL-7R signaling has been investigated across various malignancies, particularly hematologic cancers such as T-cell and B-cell acute lymphoblastic leukemia, and multiple therapeutic strategies are under development [40,41,42,43,44]. For example, monoclonal antibodies targeting IL-7Rα (CD127) are being developed to block IL-7-mediated signaling, and JAK inhibitors such as ruxolitinib suppress the downstream STAT5 pathway [40,44,45,46]. More recently, chimeric antigen receptor T cells were engineered to interfere with IL-7R signaling, opening another promising avenue [47,48,49,50]. In addition to hematologic malignancies, IL-7/IL-7R signaling is implicated in the progression of various solid tumors. In prostate cancer, high IL-7 expression correlates with poor prognosis because IL-7R activation promotes invasion and migration via the AKT/NF-κB pathway and matrix metalloproteinase (MMP) regulation [51,52]. Similarly, IL-7 enhances bladder cancer invasion through NF-κB-mediated MMP-9 expression and promotes lung cancer proliferation by upregulating cyclin D1 via the c-FOS/c-Jun pathway [53].

IRF8, a member of the interferon regulatory factor (IRF) family, plays essential roles in hematopoiesis and type I interferon signaling [54]. Although traditionally considered a tumor suppressor [20,55,56], recent studies suggest a pro-oncogenic role for IRF8, particularly in acute myeloid leukemia (AML), where elevated IRF8 expression correlates with poor prognosis [57,58,59]. Mechanistically, IRF8 promotes leukemic cell proliferation by regulating STAT3, a key component of the JAK/STAT signaling pathway. Reduced IRF8 expression decreases STAT3 and pSTAT3 levels, suppressing AML growth by downregulating cyclin A and cyclin B1, which induces S-phase arrest [57]. In addition to its role in AML, elevated IRF8 expression is associated with poor survival in lung adenocarcinoma, according to an analysis of TCGA data [60]. In clear-cell renal cell carcinoma, TAMs express high levels of IRF8, contributing to CD8^+^ T cell exhaustion [61]. High IRF8-TAM gene signatures correlate with increased T cell dysfunction, particularly in tumors with abundant CD8^+^ T cell infiltration [62]. IRF8-expressing TAMs contribute to tumor-reactive cytotoxic T lymphocyte (CTL) exhaustion by presenting tumor antigens and inducing PD-1 expression on CD8^+^ T cells. In the absence of IRF8, TAMs fail to drive T cell exhaustion, highlighting the importance of IRF8-mediated antigen presentation. High IRF8 expression in TAMs correlates with poor prognosis in CD8^+^ T cell-rich tumors, suggesting that IRF8-dependent TAM functions sustain CTL exhaustion [61]. Although IRF8 is linked to favorable outcomes in some cancers, our findings suggest that the IRF8-TAM gene signature predicts immune dysfunction in ovarian cancer, highlighting the complex and context-dependent role of IRF8 within the TME.

The leukocyte common antigen PTPRC (CD45) is a transmembrane glycoprotein broadly expressed on hematopoietic cells, except mature erythrocytes, and plays a pivotal role in immune cell activation and signaling [63]. Although PTPRC has traditionally served as a pan-leukocyte marker, emerging evidence suggests it also has prognostic and functional significance in several malignancies [64,65,66,67]. In colorectal cancer, a subset of epithelial tumor cells aberrantly expresses PTPRC, and elevated expression in those cells is linked to poor tumor regression and shorter recurrence-free survival after chemoradiotherapy [64]. Mechanistically, PTPRC enhances Wnt/β-catenin signaling by reducing β-catenin tyrosine phosphorylation, preventing its degradation, and promoting nuclear accumulation that, in turn, increases the transcription of Wnt target genes, thereby supporting CSC survival, self-renewal, and metastatic potential and highlighting a tumor-intrinsic role for PTPRC in promoting chemoresistance and progression. Similarly, in hematologic malignancies such as AML, PTPRC promotes tumor growth by localizing to lipid rafts, where it enhances GM-CSF signaling through the activation of Src family kinases [67]. These findings underscore the context-dependent and multifaceted functions of PTPRC in cancer. Our study extends this knowledge to HGSOC, where we found that high PTPRC expression is significantly associated with poor prognosis. As in colorectal cancer, PTPRC drives HGSOC progression by promoting therapy resistance and CSC-related properties.

NSG1 is involved in endocytosis, apoptosis regulation, and intracellular trafficking of membrane receptors such as AMPA-type glutamate receptors, transferrin receptors, and neurotensin receptors [68]. Through that regulation, NSG1 modulates intracellular signaling pathways that influence tumor cell proliferation, migration, and progression. NSG1 is also a transcriptional target of the tumor suppressor p53. Under genotoxic stress, p53 induces NSG1 expression, which promotes tumor suppression by facilitating the endocytic degradation of oncogenic receptors such as EGFR and integrins, thereby inhibiting pro-tumorigenic signaling [69,70,71]. Consistent with that mechanism, low NSG1 expression—as observed in our study—is associated with impaired tumor suppressor function and poor prognosis. NSG1 overexpression reduces tumor cell viability, potentially by inducing endoplasmic reticulum stress. In contrast, reduced NSG1 expression leads to decreased CHOP activation and weakened apoptotic responses to DNA damage, promoting tumor survival [69]. Beyond its tumor-intrinsic role, NSG1 also influences the TME. Low NSG1 expression correlates with reduced infiltration of CD8^+^ T cells and M1 macrophages and increased activation of M2 macrophages, thereby contributing to immune evasion and tumor progression [68].

Furthermore, our integrative correlation analysis revealed that specific DEGs, particularly *IL7R*, *IRF8*, and *PTPRC*, correlated positively with DEPs such as CHAC2, ERBIN, and LTA4H and negatively with HAVCR1 and NPY. These results suggest complex gene–protein interactions in recurrent HGSOC, many of which converge on key oncogenic pathways such as MAPK, NOTCH, VEGF, and NF-κB signaling. Our pathway enrichment analysis further highlighted the involvement of these signaling axes in recurrence, tumor progression, and treatment resistance. A schematic summary of the key signaling pathways and molecular interactions identified in this study is provided in Supplementary Figure S1.

Importantly, our multi-omics approach offers a comprehensive view of the molecular drivers that underlie recurrence and could inform the development of precision medicine strategies. Integrating genomic and proteomic profiling is essential for identifying novel druggable targets, particularly in treatment-resistant ovarian cancer. In this context, several candidate biomarkers identified in this study, particularly *IL7R*, *IRF8*, *PTPRC*, and *NSG1*, have been investigated in preclinical or clinical settings across various malignancies; the relevant translational and clinical evidence is summarized in Supplementary Table S8. Further studies should validate these candidate biomarkers in larger patient cohorts and investigate their roles in immune checkpoints and personalized treatment approaches.

Several limitations of this study must be acknowledged. First, the relatively small sample sizes in the primary/recurrent tumor and validation cohorts increase the risk of overfitting. Nevertheless, the paired analysis of primary and recurrent tumors from the same patients reduces inter-individual variability and supports the biological relevance of the observed molecular changes. In addition, only a subset of patients had matched transcriptomic and proteomic profiles, which could further limit our statistical power for cross-omics comparisons. Second, discrepancies between mRNA-based Kaplan–Meier survival data and some protein-level findings might reflect post-transcriptional regulation, as well as differences in sample types and measurement platforms. Therefore, further validation in larger external cohorts would be valuable. Third, although this study provides functional insights into *IL7R*, *IRF8*, *PTPRC*, and *NSG1*, further mechanistic studies are required to elucidate their interactions and downstream signaling pathways. Although our integrative analyses identified coordinated gene–protein expression patterns and highlighted key signaling pathways associated with recurrence, the precise molecular interactions and causal downstream mechanisms cannot be fully defined based on our current data. In particular, the DEG–DEP associations and pathway enrichments should be interpreted as context-dependent and hypothesis-generating, reflecting dynamic molecular rewiring during disease progression, rather than direct mechanistic evidence. Accordingly, further functional studies using experimental models will be required to delineate the direct regulatory interactions and downstream signaling cascades underlying these observations.

Despite those limitations, our findings offer novel insights into the mechanisms of HGSOC recurrence by characterizing key gene and protein expression changes and their interaction networks, and they thus have important implications for prognosis prediction and individualized therapeutic strategies.

## 4. Materials and Methods

### 4.1. Sample Collection and Preparation

Sixty-eight matched primary and first recurrent HGSOC tissue samples were obtained from 34 patients who were initially diagnosed and continuously treated at Gangnam Severance Hospital, Yonsei University College of Medicine, Seoul, Republic of Korea, between 2009 and 2019. Clinicopathological data, including time to first recurrence or death from ovarian cancer, were retrieved from electronic medical records. This study was approved by the Institutional Review Board of Gangnam Severance Hospital (IRB No. 3-2021-0380), and informed consent was obtained from all patients. FFPE blocks and fresh-frozen tissues were obtained from the Human Tissue Bank and the Korean Gynecologic Cancer Bank at Gangnam Severance Hospital (Resource No. HTB-P2022-3). All research procedures were conducted in accordance with all relevant ethical regulations, including the principles of the Declaration of Helsinki. All samples included in the study met the following criteria: availability of matched primary and first recurrent HGSOC samples, no history of neoadjuvant chemotherapy before the initial surgery, and sufficient histological quality of the primary and recurrent tumor samples. All specimens were reviewed by a pathologist who specializes in gynecologic oncology, and only those with tumor purity greater than 80% were included in the analyses. RNA sequencing was performed on paired FFPE samples from the primary and recurrent tumor tissues of 24 patients. Concurrently, the proteomic analysis was conducted on matched fresh-frozen tissue samples from 19 patients. Nine patients provided both FFPE and fresh-frozen tissue samples, enabling us to conduct integrated transcriptomic and proteomic analyses. The remaining 25 patients contributed only one type of sample each, precluding integrated transcriptomic and proteomic analyses (Figure 1A). The IHC analysis using TMA samples was conducted on a distinct set of paired primary and recurrent tumor tissues from 34 patients.

### 4.2. RNA Sequencing

The total RNA concentration was measured using a Quant-IT RiboGreen assay (Invitrogen, Carlsbad, CA, USA). Samples were analyzed using a TapeStation RNA ScreenTape system (Agilent Technologies, Santa Clara, CA, USA) to determine the DV200 value (percentage of RNA fragments > 200 bp). In accordance with the manufacturer’s protocol, 100 ng of total RNA was used with an Agilent SureSelect RNA direct kit to construct sequencing libraries. Total RNA was first fragmented into small pieces using divalent cations at an elevated temperature. The cleaved RNA fragments were then reverse-transcribed into first-strand cDNA using random primers, followed by second-strand cDNA synthesis. The resulting cDNA fragments underwent end repair, the addition of a single ‘A’ base, and adapter ligation. The products were subsequently purified and enriched via PCR to generate a cDNA library. An Agilent SureSelect XT human all exon V6 + UTRs kit was used as specified in the standard Agilent SureSelect target enrichment protocol. To capture human exonic regions, 250 ng of the cDNA library was combined with hybridization buffers, blocking mixes, RNase block, and 5 µL of the SureSelect all exon capture library. RNase block is an RNase inhibitor included in the SureSelect reagents and was added to prevent RNase-mediated degradation during the hybridization step; no RNase digestion/treatment was performed. Hybridization to the capture baits was performed at 65 °C using a thermal cycler with a heated lid set at 105 °C for 24 h. The captured library was then washed and subjected to a second round of PCR amplification.

The final purified product was quantified using a KAPA library quantification kit for Illumina sequencing platforms in accordance with the qPCR quantification protocol guide (KAPA BIOSYSTEMS, Wilmington, MA, USA, #KK4854). Its quality was assessed using the TapeStation D1000 ScreenTape system (Agilent Technologies, #5067-5582). The indexed libraries were submitted for sequencing on an Illumina NovaSeq platform (Illumina, Inc., San Diego, CA, USA), and paired-end (2 × 100 bp) sequencing was performed by Macrogen, Inc. (Seoul, Republic of Korea).

### 4.3. Data Preprocessing and Differential Expression Analysis of RNA Sequencing Data

Low-quality and adapter sequences were trimmed using TrimGalore (version 0.6.10) with a quality score cutoff of 20 [72]. Subsequently, the STAR 2-pass method was used to align the raw data with the human reference genome (GRCh38.p14) via the STAR aligner, and the gencode v47 annotation file was used for gene annotation [73]. The differential expression analysis was performed with the R package DESeq2 (version 1.42.0) to extract the magnitude (log2FC) and significance (*p*-value) of the gene expression values between groups [74]. The *p*-value was adjusted using the Benjamini–Hochberg procedure, and genes with an FDR < 0.01 and |log2FC| > 1 were defined as DEGs. Gene expression was visualized using a volcano plot generated with the R package ggrepel (version 0.9.6) and a heatmap produced with the R package pheatmap (version 1.0.12).

### 4.4. Ingenuity Pathway Analysis

The complete list of DEGs was uploaded to the IPA platform for candidate molecule analysis. In this network analysis, focus molecules were identified based on their documented interactions with other molecules in the QIAGEN Knowledge Base (QIAGEN, Germantown, MD, USA). Networks were constructed by maximizing the interconnectedness of the focus molecules, and additional molecules from the QIAGEN Knowledge Base were incorporated to merge smaller networks into larger ones. Each network was restricted to a maximum of 35 molecules to ensure clarity. Network significance was assessed using Fisher’s exact test, with scores calculated as the negative base-10 logarithm of the *p*-value, so higher scores denote greater statistical significance of the observed relationships.

### 4.5. Pathway Enrichment Analysis

For the canonical pathway analysis using IPA, the ratio was determined using an in-house script based on the formula.

Ratio = (N of detected genes in pathway)/(N of all genes in pathway)

The R package clusterProfiler (version 4.12.0) was used for the ORA [75]. Reference gene sets were sourced from the KEGG database and GO: BP gene sets [76,77]. Multiple testing correction was performed using the Benjamini–Hochberg method, with gene sets considered statistically significant if the FDR was less than 0.05. For those gene sets, the ratio was calculated in the same way: as the proportion of DEGs that overlapped with the gene set, relative to the total number of genes in that gene set.

### 4.6. Survival Analysis

Survival differences between the high and low expression groups were analyzed using the survival (version 3.5.8) and survminer (version 0.4.9) packages in R [78,79]. The optimal cut-point for gene expression was determined using the surv_cutpoint and surv_categorize functions. Then, Kaplan–Meier survival curves were generated using the survfit and ggsurvplot functions to evaluate the survival probabilities in patient groups stratified by high and low expression. Genes with log-rank *p*-values less than 0.01 were considered statistically significant.

### 4.7. Tissue Microarray and Immunohistochemistry

TMA blocks were constructed using FFPE donor blocks from the paired primary and recurrent tumors of 34 ovarian cancer patients. This paired TMA cohort was an independent validation set that had no overlap with the RNA-seq and proteomics cohorts. Each tissue core had a diameter of 1.5 mm, and the TMA blocks were sectioned into 5 µm thick slices using a rotary microtome. The TMA sections were incubated at 65 °C for 20 min; deparaffinized in xylene for 15 min; and transferred to 100% ethanol, 90% ethanol, and 70% ethanol for 5 min each. The sections were then rinsed with deionized water. For antigen retrieval, the sections were subjected to heat-mediated pretreatment with 10 mM citrate buffer (pH 6.0 or 9.0) for 10 min in a microwave. The sections were then immersed in methanol containing 3% hydrogen peroxide for 10 min, followed by incubation with primary antibodies for 120 min. The primary antibodies used were IL7R (Abcam, Cambridge, UK, #ab259806), IRF8 (Cell Signaling, Danvers, MA, USA, #83413), GPR68 (Abcam, #ab61420), PTPRC (Cell Signaling, #13917), WDFY4 (Abcam, #ab122661), and NSG1 (Invitrogen, #PA5-36497). The secondary antibody, EnVision™ rabbit/mouse reagent (DAKO, Santa Clara, CA, USA, #K5007), was applied for 60 min. Sections were visualized with 3,3-diaminobenzidine tetrachloride (DAKO, #K3468) and counterstained with hematoxylin (DAKO, #S3309) for 5 min. All procedures were carried out at room temperature (25 °C).

### 4.8. Evaluation of Immunohistochemistry Staining

Stained TMA sections were scanned at 400× optical magnification using an Axioscan 7 microscope slide scanner (ZEISS, Oberkochen, Germany). The scanned images were analyzed using QuPath 0.4.4 (University of Edinburgh, Edinburgh, UK), a quantitative pathology and bioimage analysis software. Representative images were captured at 2.5× and 50× digital magnification within QuPath. Each TMA core was designated as a region of interest for analysis, and automated cell segmentation was performed using the default algorithm in QuPath, with parameter adjustments. The algorithm classified cells into stained and unstained populations, and the percentage of stained cells relative to the total cell count was quantified on a scale of 0 to 100.

### 4.9. Proteomic Assay Data Generation

The fresh-frozen tissues were transferred to bead tubes on ice and lysed using T-PER™ buffer (Thermo Scientific™, Rockford, IL, USA) with protease inhibitors. Homogenization was performed using a FastPrep-24™ classic homogenizer (MP Biomedicals, Inc., Santa Ana, CA, USA), followed by multiple cycles of centrifugation and supernatant collection. Protein concentrations were measured using bovine serum albumin, and all samples were adjusted to a uniform concentration. The prepared samples were analyzed using the Olink Explore Oncology II panel (Olink Proteomics AB, Uppsala, Sweden), which comprises 386 oncology-related antibodies. This technology relies on the precise binding of target proteins to antibody probes, which are tagged with dual oligonucleotides. The subsequent DNA sequences were quantitatively detected through microfluidic RT-qPCR amplification of the oligonucleotides. Expression values were normalized using experimental controls, and the results are presented as normalized, log2-transformed protein expression values. This experiment was conducted by DNA Link (Seoul, Republic of Korea).

### 4.10. Data Preprocessing and Analysis of Proteomics Data

NPX values were used for the differential expression analysis of proteins that passed quality control. The comparison was performed using empirical Bayes-moderated *t*-tests implemented through the limma (version 3.56.0) R package [80]. Proteins with a *p*-value less than 0.05 were considered to be DEPs. Co-expression was analyzed using Spearman’s rank correlation coefficients, and a correlation with a *p*-value less than 0.05 was considered significant.

## 5. Conclusions

Our integrative multi-omics analysis reveals novel molecular mechanisms driving HGSOC recurrence, identifying IL7R, IRF8, PTPRC, and NSG1 as potential biomarkers and therapeutic targets. By contributing to immune evasion, tumor progression, and treatment resistance, these genes illuminate critical pathways in HGSOC recurrence, underscoring their potential as both prognostic markers and therapeutic targets. Future research should validate these findings in larger cohorts and further explore their roles in modulating the TME and immune response. Additionally, combination therapies targeting these molecules, alongside immune checkpoint inhibitors or chemotherapy, might improve clinical outcomes for recurrent HGSOC patients.

## Figures and Tables

**Table 1:** Clinicopathological characteristics of enrolled 34 patients.

Characteristics	N (Median)	(%)
All cases	34	100
Age (years)	(53) range 41–69	
CA125 at diagnosis (U/mL)	(639.5) range 50.0–20,388.1	
FIGO staging		
I, II	1	2.9
III	28	82.4
IV	5	14.7
Histological grade		
G2	6	17.6
G3	26	76.5
unknown	2	5.9
BRCA status		
BRCA wild type	21	61.8
BRCA1 mutation	4	11.8
BRCA2 mutation	2	5.9
unknown	7	20.6

## Data Availability

The transcriptomic data produced in this study are publicly available through the Gene Expression Omnibus (GEO) database with accession number GSE295041.

## Supplementary Materials

The following supporting information can be downloaded at https://www.mdpi.com/article/10.3390/ijms27020948/s1. References [81,82,83,84,85,86,87,88,89,90,91,92,93,94] are cited in Supplementary Materials.

## Abbreviations

The following abbreviations are used in this manuscript:

EOC	Epithelial ovarian cancer
HGSOC	High-grade serous ovarian cancer
FFPE	Formalin-fixed paraffin-embedded samples
DEGs	Differentially expressed genes
IHC	Immunohistochemistry
TMA	Tissue microarray
FDR	False discovery rate
IPA	Ingenuity pathway analysis
ORA	Over-representation analysis
KEGG	Kyoto Encyclopedia of Genes and Genomes
GO: BP	Gene ontology biological process
DEPs	Differentially expressed proteins
TME	Tumor microenvironment
TAM	Tumor-associated macrophage
Tregs	Regulatory T cells
CSC	Cancer stem cell
MMP	Matrix metalloproteinase
IRF	Interferon regulatory factor
AML	Acute myeloid leukemia
CTL	Cytotoxic T lymphocyte
